# 3,4-Diamino­pyridinium hydrogen succinate

**DOI:** 10.1107/S1600536809021205

**Published:** 2009-06-10

**Authors:** Hoong-Kun Fun, Kasthuri Balasubramani

**Affiliations:** a X-ray Crystallography Unit, School of Physics, Universiti Sains Malaysia, 11800 Universiti Sains Malaysia, Penang, Malaysia

## Abstract

In the title compound, C_5_H_8_N_3_^+^·C_4_H_5_O_4_^−^, the pyridine N atom of the 3,4-diamino­pyridine mol­ecule is protonated. The protonated N atom participates in an N—H⋯O hydrogen bond to a succinate O atom of the singly deprotonated succinate anion. Each of the two amino groups are hydrogen-bonded to the O atoms of two different sets of succinate groups.. The crystal structure is further stabilized by O—H⋯O and C—H⋯O hydrogen bonds.

## Related literature

For background to the chemistry of substituted pyridines, see: Pozharski *et al.* (1997 ▶); Katritzky *et al.* (1996 ▶). For the use of 3,4-diamino­pyridine in Schiff base reactions, see: Opozda *et al.* (2006 ▶). For related structures, see: Opozda *et al.* (2006 ▶); Rubin-Preminger & Englert (2007 ▶); Koleva *et al.* (2007 ▶, 2008 ▶). For bond-length data, see: Allen *et al.* (1987 ▶) and for hydrogen-bond motifs, see: Bernstein *et al.* (1995 ▶). For the stability of the temperature controller used in the data collection, see: Cosier & Glazer (1986 ▶).
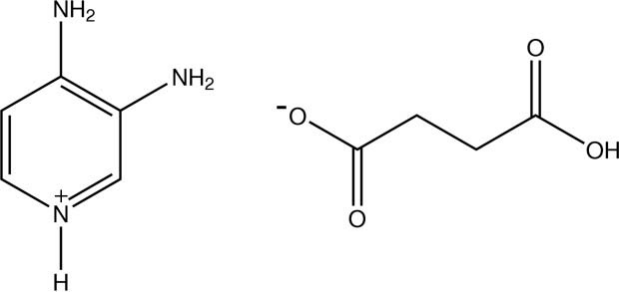


## Experimental

### 

#### Crystal data

C_5_H_8_N_3_^+^·C_4_H_5_O_4_^−^
*M* *_r_* = 227.22
Monoclinic, 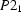

*a* = 4.9862 (2) Å
*b* = 9.5028 (3) Å
*c* = 10.4775 (3) Å
β = 93.653 (2)°
*V* = 495.45 (3) Å^3^
*Z* = 2
Mo *K*α radiation
μ = 0.12 mm^−1^
*T* = 100 K
0.41 × 0.13 × 0.08 mm

#### Data collection

Bruker APEXII CCD area-detector diffractometer
Absorption correction: multi-scan (*SADABS*; Bruker, 2005 ▶) *T*_min_ = 0.929, *T*_max_ = 0.991
9518 measured reflections
2280 independent reflections
2119 reflections with *I* > 2σ(*I*)
*R*_int_ = 0.028

#### Refinement

*R*[*F* ^2^ > 2σ(*F* ^2^)] = 0.036
*wR*(*F* ^2^) = 0.104
*S* = 1.18
2280 reflections
197 parameters
1 restraint
All H-atom parameters refined
Δρ_max_ = 0.41 e Å^−3^
Δρ_min_ = −0.24 e Å^−3^

### 

Data collection: *APEX2* (Bruker, 2005 ▶); cell refinement: *SAINT* (Bruker, 2005 ▶); data reduction: *SAINT*; program(s) used to solve structure: *SHELXTL* (Sheldrick, 2008 ▶); program(s) used to refine structure: *SHELXTL*; molecular graphics: *SHELXTL*; software used to prepare material for publication: *SHELXTL* and *PLATON* (Spek, 2009 ▶).

## Supplementary Material

Crystal structure: contains datablocks global, I. DOI: 10.1107/S1600536809021205/sj2630sup1.cif

Structure factors: contains datablocks I. DOI: 10.1107/S1600536809021205/sj2630Isup2.hkl

Additional supplementary materials:  crystallographic information; 3D view; checkCIF report

## Figures and Tables

**Table 1:** Hydrogen-bond geometry (Å, °)

*D*—H⋯*A*	*D*—H	H⋯*A*	*D*⋯*A*	*D*—H⋯*A*
O2—H9⋯O4^i^	0.99 (4)	1.53 (4)	2.4815 (14)	159 (3)
N1—H1*N*1⋯O3^ii^	0.93 (3)	1.80 (3)	2.7036 (16)	163 (3)
N2—H1*N*2⋯O2^iii^	0.77 (3)	2.36 (3)	3.0440 (17)	150 (3)
N2—H2*N*2⋯O1^iv^	0.97 (3)	2.00 (3)	2.9473 (17)	164 (3)
N3—H1*N*3⋯O1^iv^	0.82 (3)	2.15 (3)	2.9720 (18)	176 (2)
N3—H2*N*3⋯O3^v^	0.93 (2)	2.23 (2)	3.0699 (17)	149.7 (18)
C2—H2*A*⋯O3^v^	0.91 (2)	2.56 (3)	3.2907 (17)	138 (2)
C5—H5*A*⋯O2^iii^	0.84 (2)	2.59 (2)	3.1923 (18)	129.7 (19)

Symmetry codes: (i) 
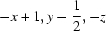
; (ii) 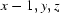; (iii) 
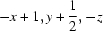
; (iv) 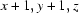; (v) 
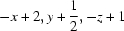
.

**Fig. 1.**
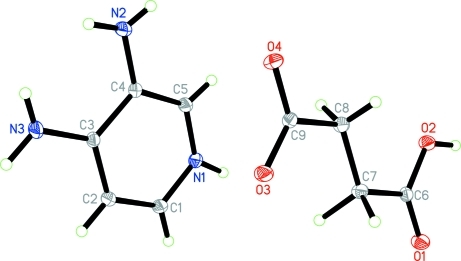
The molecular structure of (I), showing 50% probability displacement ellipsoids and the atom numbering scheme.

**Fig. 2.**
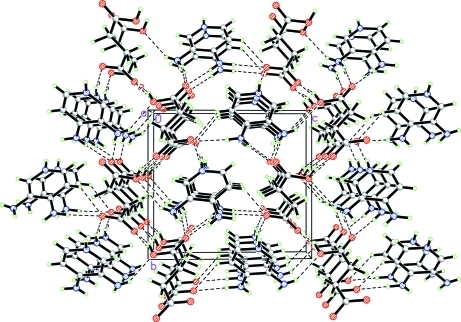
The overall three-dimensional network of (I). Dashed lines indicate hydrogen bonds.

## supplementary crystallographic information

### Comment

Pyridine and its derivatives play an important role in heterocyclic chemistry (Pozharski *et al.* 1997; Katritzky *et al.* 1996). 3,4-diaminopyridine is used as a component in Schiff base reactions (Opozda *et al.* 2006). The crystal structure of 3,4-diaminopyridine (Rubin-Preminger & Englert, 2007), 3,4-diaminopyridinium hydrogen squarate (Koleva *et al.*, 2007) and 3,4-diaminopyridinium hydrogen tartarate (Koleva *et al.*, 2008) have been reported in the literature. Since our aim is to study some interesting hydrogen-bonding interactions, the synthesis and structure of the title compound (I) is presented here.

The asymmetric unit of (I) (Fig 1), contains a protonated 3,4-diaminopyridinium cation and a hydrogen succinate anion. The bond lengths (Allen *et al.*, 1987) and angles are normal. In the 3,4-diaminopyridinium cation, protonation of the N1 atom leads to a slight increase in the C1—N1—C5 angle to 121.56 (12)°, compared to 115.69 (19)° in 3,4-diaminopyridine (Rubin-Preminger & Englert, 2007). The 3,4-diaminopyridinium cation is planar, with a maximum deviation of 0.0070 (15)Å for atom C4.

In the crystal packing (Fig. 2), the protonated N1 atom is hydrogen bonded to the carboxylate oxygen atom of O3 through N—H···O hydrogen bonds. The two amino groups (N2 and N3) are involved in the hydrogen bonding *via* N—H···O H-bonds with hydrogen succinate oxygen atom (O1) to form an *R*^1^_2_(7) ring motif  (Bernstein *et al.*, 1995). The N3 amino group and ring carbon atom (C2) are both hydrogen-bonded to the carboxylate oxygen atom (O3) to form an *R*^1^_2_(6) ring motif. The molecules are further connected *via* O—H···O hydrogen bonds forming a 3-D network (Table 1 and Fig 2).

### Experimental

Hot methanol solutions (20 ml) of 3,4-diaminopyridine (27 mg, Aldrich) and succinic acid (29 mg, Merck) were mixed and warmed for 5 minutes. The resulting solution was allowed to cool slowly at room temperature. Crystals of (I) appeared from the mother liquor after a few days.

### Refinement

All the H aroms were located from the difference Fourier map [N–H = 0.93 (3)–0.97 (3) Å, C–H = 0.84 (2)–1.13 (3)Å & O–H = 0.99 (4) Å] and allowed to refine freely. In the absence of significant anomalous scattering effects, 1862 Friedel pairs were merged.

### Crystal data

**Table:**

C_5_H_8_N_3_^+^·C_4_H_5_O_4_^−^	*F*(000) = 240
*M**_r_* = 227.22	*D*_x_ = 1.523 Mg m^−^^3^
Monoclinic, *P*2_1_	Mo *K*α radiation, λ = 0.71073 Å
Hall symbol: P 2yb	Cell parameters from 4153 reflections
*a* = 4.9862 (2) Å	θ = 2.9–38.4°
*b* = 9.5028 (3) Å	µ = 0.12 mm^−^^1^
*c* = 10.4775 (3) Å	*T* = 100 K
β = 93.653 (2)°	Block, colourless
*V* = 495.45 (3) Å^3^	0.41 × 0.13 × 0.08 mm
*Z* = 2	

### Data collection

**Table:**

Bruker APEXII CCD area-detector diffractometer	2280 independent reflections
Radiation source: fine-focus sealed tube	2119 reflections with *I* > 2σ(*I*)
graphite	*R*_int_ = 0.028
φ and ω scans	θ_max_ = 35.0°, θ_min_ = 2.0°
Absorption correction: multi-scan (*SADABS*; Bruker, 2005)	*h* = −8→8
*T*_min_ = 0.929, *T*_max_ = 0.991	*k* = −15→15
9518 measured reflections	*l* = −16→16

### Refinement

**Table:**

Refinement on *F*^2^	Primary atom site location: structure-invariant direct methods
Least-squares matrix: full	Secondary atom site location: difference Fourier map
*R*[*F*^2^ > 2σ(*F*^2^)] = 0.036	Hydrogen site location: inferred from neighbouring sites
*wR*(*F*^2^) = 0.104	All H-atom parameters refined
*S* = 1.18	*w* = 1/[σ^2^(*F*_o_^2^) + (0.0624*P*)^2^ + 0.022*P*] where *P* = (*F*_o_^2^ + 2*F*_c_^2^)/3
2280 reflections	(Δ/σ)_max_ < 0.001
197 parameters	Δρ_max_ = 0.41 e Å^−^^3^
1 restraint	Δρ_min_ = −0.24 e Å^−^^3^

### Special details

**Table:**

Experimental. The crystal was placed in the cold stream of an Oxford Cryosystems Cobra open-flow nitrogen cryostat (Cosier & Glazer, 1986) operating at 100.0 (1) K.
Geometry. All s.u.'s (except the s.u. in the dihedral angle between two l.s. planes) are estimated using the full covariance matrix. The cell s.u.'s are taken into account individually in the estimation of s.u.'s in distances, angles and torsion angles; correlations between s.u.'s in cell parameters are only used when they are defined by crystal symmetry. An approximate (isotropic) treatment of cell s.u.'s is used for estimating s.u.'s involving l.s. planes.
Refinement. Refinement of F^2^ against ALL reflections. The weighted R-factor wR and goodness of fit S are based on F^2^, conventional R-factors R are based on F, with F set to zero for negative F^2^. The threshold expression of F^2^ > σ(F^2^) is used only for calculating R-factors(gt) etc. and is not relevant to the choice of reflections for refinement. R-factors based on F^2^ are statistically about twice as large as those based on F, and R- factors based on ALL data will be even larger.

### Fractional atomic coordinates and isotropic or equivalent isotropic displacement parameters (Å^2^)

**Table:**

	*x*	*y*	*z*	*U*_iso_*/*U*_eq_	
O1	0.2865 (2)	−0.15266 (13)	0.23443 (10)	0.0166 (2)	
O2	0.2994 (2)	−0.04711 (12)	0.04363 (10)	0.0145 (2)	
O3	1.0339 (2)	0.20905 (12)	0.27633 (10)	0.0148 (2)	
O4	1.0369 (2)	0.31053 (11)	0.08455 (10)	0.01284 (19)	
C6	0.3844 (3)	−0.06666 (14)	0.16351 (13)	0.0109 (2)	
C7	0.6179 (3)	0.02567 (15)	0.20940 (13)	0.0127 (2)	
C8	0.7020 (3)	0.13495 (15)	0.11407 (13)	0.0120 (2)	
C9	0.9402 (3)	0.22422 (14)	0.16284 (12)	0.0100 (2)	
N1	0.4164 (2)	0.40451 (13)	0.33423 (12)	0.0123 (2)	
N2	0.8257 (3)	0.65927 (15)	0.17427 (12)	0.0172 (2)	
N3	1.0198 (3)	0.68665 (15)	0.43411 (12)	0.0148 (2)	
C1	0.5088 (3)	0.41464 (15)	0.45697 (13)	0.0128 (2)	
C2	0.7131 (3)	0.50705 (15)	0.49097 (13)	0.0123 (2)	
C3	0.8247 (3)	0.59224 (14)	0.39891 (13)	0.0108 (2)	
C4	0.7264 (3)	0.57828 (14)	0.26856 (13)	0.0113 (2)	
C5	0.5199 (3)	0.48313 (15)	0.24195 (13)	0.0121 (2)	
H9	0.149 (6)	−0.108 (4)	0.012 (3)	0.059 (11)*	
H1N1	0.270 (6)	0.343 (4)	0.329 (3)	0.039 (8)*	
H1N2	0.801 (5)	0.635 (3)	0.105 (3)	0.026 (7)*	
H2N2	0.992 (6)	0.706 (4)	0.202 (3)	0.036 (7)*	
H1N3	1.097 (5)	0.734 (3)	0.382 (2)	0.019 (6)*	
H2N3	1.073 (4)	0.697 (3)	0.520 (2)	0.018 (6)*	
H1A	0.426 (4)	0.362 (3)	0.516 (2)	0.020 (6)*	
H2A	0.770 (4)	0.517 (3)	0.574 (2)	0.018 (5)*	
H5A	0.458 (5)	0.465 (3)	0.167 (2)	0.015 (5)*	
H7A	0.570 (5)	0.073 (3)	0.304 (3)	0.029 (7)*	
H7B	0.758 (6)	−0.037 (4)	0.247 (3)	0.043 (8)*	
H8A	0.757 (5)	0.094 (3)	0.043 (3)	0.025 (6)*	
H8B	0.570 (4)	0.203 (3)	0.097 (2)	0.017 (5)*	

### Atomic displacement parameters (Å^2^)

**Table:**

	*U*^11^	*U*^22^	*U*^33^	*U*^12^	*U*^13^	*U*^23^
O1	0.0190 (5)	0.0174 (5)	0.0134 (4)	−0.0080 (4)	0.0009 (4)	0.0021 (4)
O2	0.0170 (4)	0.0147 (4)	0.0111 (4)	−0.0043 (4)	−0.0033 (3)	0.0011 (3)
O3	0.0168 (4)	0.0152 (5)	0.0118 (4)	−0.0047 (4)	−0.0039 (3)	0.0026 (4)
O4	0.0121 (4)	0.0130 (4)	0.0132 (4)	−0.0020 (3)	−0.0006 (3)	0.0040 (3)
C6	0.0115 (5)	0.0107 (5)	0.0107 (5)	−0.0012 (4)	0.0015 (4)	−0.0015 (4)
C7	0.0130 (5)	0.0131 (5)	0.0116 (5)	−0.0046 (4)	−0.0015 (4)	0.0019 (4)
C8	0.0113 (5)	0.0129 (5)	0.0113 (5)	−0.0038 (4)	−0.0020 (4)	0.0006 (4)
C9	0.0094 (5)	0.0095 (5)	0.0109 (5)	0.0000 (4)	−0.0006 (4)	0.0004 (4)
N1	0.0129 (5)	0.0114 (5)	0.0124 (5)	−0.0025 (4)	−0.0001 (4)	0.0000 (4)
N2	0.0227 (6)	0.0187 (6)	0.0103 (5)	−0.0088 (5)	0.0012 (4)	0.0007 (4)
N3	0.0164 (5)	0.0160 (5)	0.0119 (5)	−0.0066 (4)	0.0002 (4)	−0.0018 (4)
C1	0.0143 (5)	0.0130 (5)	0.0112 (5)	−0.0027 (4)	0.0016 (4)	0.0014 (4)
C2	0.0142 (5)	0.0128 (5)	0.0100 (5)	−0.0015 (4)	0.0005 (4)	0.0003 (4)
C3	0.0110 (5)	0.0105 (5)	0.0108 (5)	−0.0013 (4)	0.0000 (4)	−0.0013 (4)
C4	0.0129 (5)	0.0105 (5)	0.0104 (5)	−0.0016 (4)	0.0003 (4)	−0.0005 (4)
C5	0.0127 (5)	0.0130 (5)	0.0105 (5)	−0.0022 (4)	−0.0004 (4)	−0.0003 (4)

### Geometric parameters (Å, °)

**Table:**

O1—C6	1.2265 (18)	N1—H1N1	0.93 (3)
O2—C6	1.3130 (17)	N2—C4	1.3695 (19)
O2—H9	0.99 (4)	N2—H1N2	0.77 (3)
O3—C9	1.2578 (16)	N2—H2N2	0.97 (3)
O4—C9	1.2760 (16)	N3—C3	1.3569 (18)
C6—C7	1.5118 (19)	N3—H1N3	0.82 (3)
C7—C8	1.5183 (19)	N3—H2N3	0.93 (2)
C7—H7A	1.13 (3)	C1—C2	1.3749 (19)
C7—H7B	0.98 (3)	C1—H1A	0.91 (2)
C8—C9	1.5213 (18)	C2—C3	1.4013 (19)
C8—H8A	0.90 (3)	C2—H2A	0.91 (2)
C8—H8B	0.93 (2)	C3—C4	1.4275 (18)
N1—C1	1.3414 (18)	C4—C5	1.3853 (18)
N1—C5	1.3501 (18)	C5—H5A	0.84 (2)
			
C6—O2—H9	116 (2)	C4—N2—H1N2	118 (2)
O1—C6—O2	123.87 (13)	C4—N2—H2N2	112.4 (17)
O1—C6—C7	121.47 (12)	H1N2—N2—H2N2	121 (2)
O2—C6—C7	114.66 (12)	C3—N3—H1N3	122.4 (17)
C6—C7—C8	115.28 (11)	C3—N3—H2N3	119.1 (16)
C6—C7—H7A	107.9 (14)	H1N3—N3—H2N3	119 (2)
C8—C7—H7A	113.1 (15)	N1—C1—C2	119.84 (13)
C6—C7—H7B	107 (2)	N1—C1—H1A	117.7 (15)
C8—C7—H7B	117.4 (18)	C2—C1—H1A	122.4 (15)
H7A—C7—H7B	94 (2)	C1—C2—C3	120.72 (12)
C7—C8—C9	113.76 (10)	C1—C2—H2A	119.8 (16)
C7—C8—H8A	111.0 (18)	C3—C2—H2A	119.4 (16)
C9—C8—H8A	104.5 (17)	N3—C3—C2	120.27 (12)
C7—C8—H8B	112.4 (14)	N3—C3—C4	121.19 (12)
C9—C8—H8B	101.8 (15)	C2—C3—C4	118.54 (11)
H8A—C8—H8B	113 (2)	N2—C4—C5	121.32 (12)
O3—C9—O4	123.29 (12)	N2—C4—C3	121.34 (12)
O3—C9—C8	119.24 (12)	C5—C4—C3	117.30 (12)
O4—C9—C8	117.47 (11)	N1—C5—C4	122.02 (12)
C1—N1—C5	121.56 (12)	N1—C5—H5A	114.7 (16)
C1—N1—H1N1	108.8 (17)	C4—C5—H5A	123.1 (16)
C5—N1—H1N1	129.4 (17)		
			
O1—C6—C7—C8	−175.22 (13)	C1—C2—C3—C4	−1.6 (2)
O2—C6—C7—C8	5.09 (18)	N3—C3—C4—N2	0.2 (2)
C6—C7—C8—C9	−179.08 (12)	C2—C3—C4—N2	179.47 (13)
C7—C8—C9—O3	−4.90 (18)	N3—C3—C4—C5	−177.55 (14)
C7—C8—C9—O4	174.38 (12)	C2—C3—C4—C5	1.74 (19)
C5—N1—C1—C2	0.1 (2)	C1—N1—C5—C4	0.0 (2)
N1—C1—C2—C3	0.7 (2)	N2—C4—C5—N1	−178.70 (13)
C1—C2—C3—N3	177.65 (14)	C3—C4—C5—N1	−1.0 (2)

### Hydrogen-bond geometry (Å, °)

**Table:**

*D*—H···*A*	*D*—H	H···*A*	*D*···*A*	*D*—H···*A*
O2—H9···O4^i^	0.99 (4)	1.53 (4)	2.4815 (14)	159 (3)
N1—H1N1···O3^ii^	0.93 (3)	1.80 (3)	2.7036 (16)	163 (3)
N2—H1N2···O2^iii^	0.77 (3)	2.36 (3)	3.0440 (17)	150 (3)
N2—H2N2···O1^iv^	0.97 (3)	2.00 (3)	2.9473 (17)	164 (3)
N3—H1N3···O1^iv^	0.82 (3)	2.15 (3)	2.9720 (18)	176 (2)
N3—H2N3···O3^v^	0.93 (2)	2.23 (2)	3.0699 (17)	149.7 (18)
C2—H2A···O3^v^	0.91 (2)	2.56 (3)	3.2907 (17)	138 (2)
C5—H5A···O2^iii^	0.84 (2)	2.59 (2)	3.1923 (18)	129.7 (19)

Symmetry codes: (i) −*x*+1, *y*−1/2, −*z*; (ii) *x*−1, *y*, *z*; (iii) −*x*+1, *y*+1/2, −*z*; (iv) *x*+1, *y*+1, *z*; (v) −*x*+2, *y*+1/2, −*z*+1.
